# Improved Biochemical Parameters in Patients Who Undergo Early Resection in Isolated Ileocaecal Crohn's Disease

**DOI:** 10.1155/2017/4396573

**Published:** 2017-07-10

**Authors:** Mirzafaraz Saeed, Hari Hullur, Amro Salem, Abbas Ali, Yousif Sahib, Mobeen Ashfaq

**Affiliations:** ^1^Department of Surgery, King Hamad University Hospital, Busaiteen, Bahrain; ^2^King Hamad University Hospital, Busaiteen, Bahrain

## Abstract

**Introduction:**

The aim of this study is to evaluate the outcome of introduction of early surgery in the course of isolated ileocecal Crohn's disease, where there is no absolute indication of surgery.

**Methods:**

Observational study involving patients with isolated ileocecal Crohn's disease who underwent early surgical resection (within one year of the presentation of the hospital). A complete blood count, ESR, and CRP were done and compared between the preoperative value, 1st postoperative visit (3-4 weeks), and last follow-up visit. Statistical analysis was done using Analysis of Variance (ANOVA) to compare the different figures.

**Results:**

There was a statistically significant increase in the hemoglobin levels between preoperative, postoperative, and long-term follow-up and a significant decrease in leukocyte count between the pre- and postoperative values (*F* = 19.8, *p* < 0.001 and *F* = 8.9, *p* = 0.002, resp.). Similarly, the ESR and CRP values were decreased significantly at long-term follow-up (*F* = 8.5, *p* = 0.019 and *F* = 8.3, *p* = 0.013, resp.).

**Conclusion:**

Early surgical resection in isolated ileocaecal Crohn's disease achieved significant biochemical improvements. These successful results in this small number of patients indicate that early surgical intervention may provide better outcomes. These initial results encourage larger and comparative studies of long-term results versus long-term use of biological agents.

## 1. Introduction

Crohn's disease is an inflammatory bowel disease subtype that involves transmural inflammation of the gastrointestinal tract. This inflammation can be present anywhere from the mouth to the perianal area. Ileocaecal Crohn's disease, the focus of this particular study, is where the focal intensity of the disease activity involves the terminal ileum and proximal caecum. While Crohn's disease also can be accompanied by a myriad of extracolonic manifestations, the majority of symptoms related to colonic involvement can include crampy abdominal pain and fluctuant nonbleeding diarrhea, with associated symptoms including fever, weight loss, presence of fistulas, and malabsorptive symptoms (such as watery diarrhea and steatorrhea).

While there are several medical therapies to choose from, surgical management, usually with intestinal resection, is still considered an important treatment modality due to the nature of Crohn's disease symptoms and complications, namely, perforation, obstruction, and strictures, being so difficult to adequately control [1, 2]. Ileocaecal resection is one of the surgical modalities, used particularly for ileocaecal Crohn's disease, and is considered to be the most effective at improving quality of life [3, 4].

In addition to the use of surgical intervention, certain blood tests are important during the overall investigation process of Crohn's disease in both the preoperative and postoperative phases. The patient's hemoglobin (Hb), White Blood Cell (WBC) count, C-Reactive Protein (CRP), and Erythrocyte Sedimentation Rate (ESR) are all important biochemical tests that help to correlate the extent of the disease activity [5–7]. Subsequently, these indicators help in predicting which patients will receive the most benefit from treatment [8, 9].

The aim of this particular study is to examine patients with ileocaecal Crohn's disease and to study their postoperative status. This will be done by evaluation of the above listed biochemical tests, to determine if early surgical intervention, in the form of hemicolectomy or ileocaecal resection, is beneficial.

## 2. Materials and Methods

### 2.1. Criteria for Inclusion and Exclusion

A cohort of patients who had isolated ileocaecal Crohn's disease and underwent early elective surgical resection, defined as surgery within one year of their presentation to the hospital, were initially selected.

The patients that presented during the study's time frame but definitely required surgical intervention or had had previous surgeries due to Crohn's disease were excluded.

From the available sample of patients, those who previously had no surgeries due to Crohn's disease and those in whom surgery was mandatorily indicated were selected (*n* = 14). Due to insufficient data, some patients were excluded (*n* = 3). A total of 11 patients, operated on between 2009 and 2012, met this criterion and were thus included in the study (*n* = 11).

### 2.2. Data Extraction

The aim of this study was to investigate the improvement in the biochemical markers in patients undergoing early elective ileocaecal resection, defined as surgery within 1 year of initial presentation to the hospital for the disease.

The main parameters measured were pre- and postoperative values of Hb, WBC, CRP, and ESR. These biochemical markers were measured preoperatively, at the 1st postoperative visit (3-4 weeks), and at the long-term follow-up visit. The mean duration from surgery to the long-term follow-up visit was 13.5 months.

The mentioned values were obtained from the patients' medical record database at King Fahad Specialist Hospital in Dammam, Saudi Arabia. Additional data obtained included age and gender of the patient, indication for surgery, date of diagnoses and date of surgery, hospital stay, and short and long-term complications postoperatively.

### 2.3. Outcome Measurement and Analysis

The measured outcomes were postoperative Hb, WBC, CRP, and ESR values measured 3-4 weeks postoperatively and at a long-term follow-up appointment, compared to their preoperative values. These parameters were analyzed for each individual patient and also as a collective mean for all patients in this study. Statistical analysis was done using SPSS version 23, and Analysis of Variance (ANOVA) was used to compare the different figures. The results of the statistical analysis are described and discussed below in Discussion.

## 3. Results

**Figure 1:**
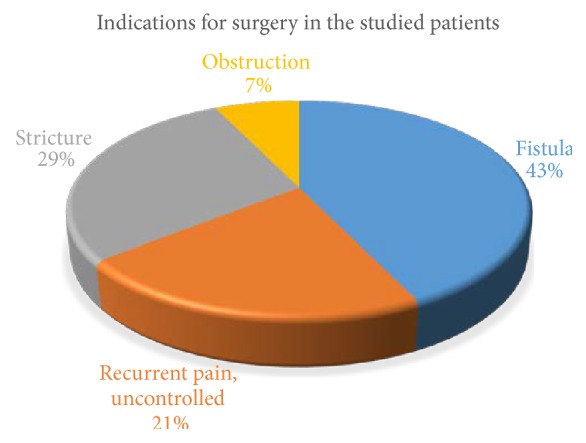
Indications for surgery in the study's sampled patients.

This study initially recruited 14 patients (which had initially 8 males and 6 females); however 3 patients were excluded from the final study due to insufficient data. The mean age of the patients recruited was 28.2 years, with the oldest patient being 47 years of age and the youngest being 16 years of age. The most common initial symptom of presentation was abdominal pain. The most common indication for surgery was fistula formation (Figure 1). The average duration from the first presentation to the surgery was 6 months, with a maximum of 12 months. A total of 14 patients were operated upon (including the 3 that were eventually excluded); 4 of those had undergone a right hemicolectomy and 10 an ileocaecal resection. There was an average follow-up time of 13.5 months with a maximum follow-up of 2 years (see Table 1).

It is important to note that the same team operated on all the patients in this study. Postoperatively, approximately 14% had had early complications after the surgery, with leakage and wound infection being frequent. None of patients had developed late complications during the course of follow-up. No patients required further surgical intervention and control of symptoms was achieved in all the patients.

**Figure 2:**
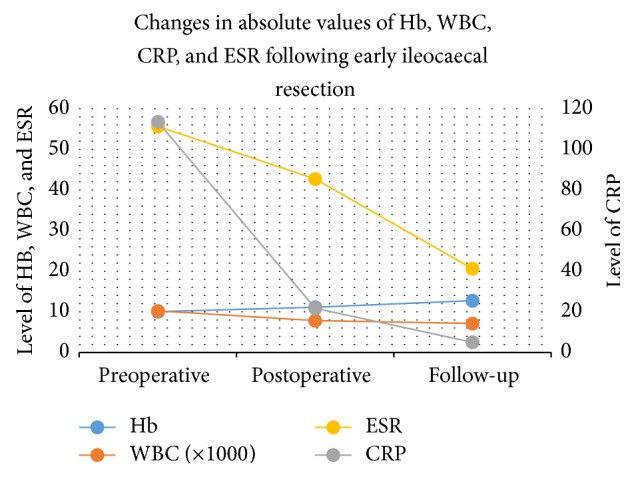
Comparative chart displaying the improvements in the values of the measured values.

As mentioned earlier, the purpose of this study was to measure the improvement in the biochemical parameters. The primary outcome measures were serum Hb, WBC, CRP, and ESR, measured preoperatively, 3-4 weeks postoperatively, and at long-term follow-up.  Table 2 and Figure 2 summarize the averages of the absolute values that were obtained. In order to better measure the change in the biochemical parameters, a percentage difference was calculated.

**Figure 3:**
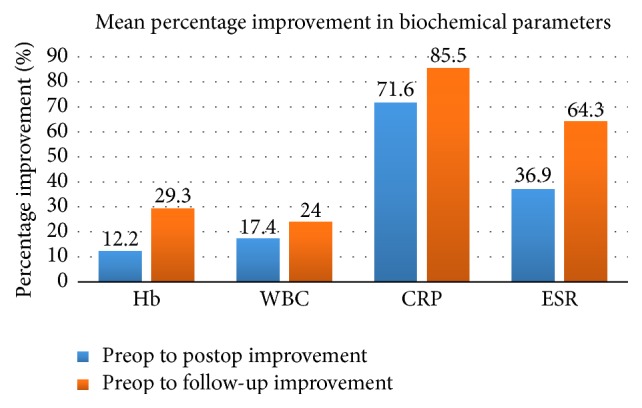
Mean percentage improvements in all the measured parameters.

The percentage improvement of the biochemical parameters was calculated between preoperative and postoperative values. A comparison was also made between preoperative and follow-up values, as shown in Table 3. CRP values improved, with values showing a 71.5% drop from preoperative to postoperative values. Results also indicated that CRP values dropped by 85.5% from preoperative to postoperative measurement. ESR values during the interval showed a percentage drop of 36.9% and 64.3%, again indicating an improvement in this biochemical parameter. Hemoglobin levels improved by 12.2% postoperatively and by 29.3% at follow-up. The improvements in WBC at the same intervals were 17.4% and 24.0%, respectively (Figure 3).

## 4. Discussion

Recent trends in the management of Crohn's disease have predicated that the focus of treatment largely returns to the domain of the medical gastroenterologists in the long-contested back-and-forth between physicians and surgeons. Due to the successes of steroid therapy and newer biologic agents, surgical intervention has mostly been relegated to being an option of last resort. Our data presented here suggest with statistical significance that, in cases of ileocaecal Crohn's disease, surgical intervention,* at an early stage*, may be warranted. There is an amorphous body of evidence that supports this claim and is discussed further below.

In 2011, Latella et al. published a viewpoint piece on the value of early surgery in Crohn's disease [10]. They state that “surgery is an almost inevitable event in the natural history of ileal or ileocolic disease” and thus call for data on the outcomes of early resection. Our study addresses that need and found a statistically significant increase in the hemoglobin levels between preoperative, postoperative, and long-term follow-up measurements and a significant decrease in leukocyte count between the same measurement points (*F* = 19.8, *p* < 0.001 and *F* = 8.9, *p* = 0.002, resp.). Similarly, the ESR and CRP values decreased significantly (*F* = 8.5, *p* = 0.019 and *F* = 8.3, *p* = 0.013, resp.). The various means and their respective *T*-values and *p* values are summarized in Table 4.

Building on the assertion that surgical intervention is inevitable in ileocaecal CD is the evidence that 44%, 61%, and 71% of patients undergo resection at 1, 5, and 10 years [11]. Furthermore, data elsewhere suggests that patients who undergo ileocaecal resection only require repeat resection in roughly 31% of cases after 10 years [12]. Altogether, the above points coalesce to the following conclusion: surgery is an almost inevitable event in the natural history of ileal or ileocolic Crohn's disease, and since most patients will undergo said surgery, the question is whether to perform it early or late in the disease course. Traditional approaches have always preferred the last resort approach, but our data here show that there are demonstrable benefits to performing early resection. This is particularly valid since the rates of repeat surgery are also low at long-term follow-up [13]. Furthermore, it has been shown that early surgery lengthens the period of clinical remission compared to late surgery, further supporting the thesis [14]. In addition, another study found* no* difference in reoperation rates with early resection, countering the study referenced previously above [15].

There is some resistance and need for query with regard to the above. The ECCO consensus on diagnosis and management of Crohn's disease currently recommends primary surgery* after* failure of medical treatment [16]. Adding to this, biologic agents, particularly anti-TNFa antibodies, reduce the need for surgery and have been increasing in popularity and use. Despite a paucity of long-term studies using these agents, they may well prevail as the superior method of controlling Crohn's disease so as to avoid the risks of major surgery, including those of anaesthesia (though biologics still entail significant risks).

Lastly, the basic but vital consideration of the size of this study warrants further investigation into the subject with a larger patient population. Despite finding statistically significant results, a larger study is needed to increase the power of the study and its applicability to the wider population. In addition, more study into the outcomes of biologic therapy when directly compared to early surgery is needed, because should biologics fulfill their growing promise with long-term outcomes superior to those of early surgery, this may obviate the findings presented here.

## 5. Conclusion

In conclusion, the study presented here finds statistically significant improvements in Hb, CRP, ESR, and WBC, postoperatively and at long-term follow-up in patients with ileocaecal Crohn's disease who underwent early ileocaecal resection. There is evidence to suggest that surgery is highly likely in the ileocaecal subset of CD patients; therefore our evidence helps to guide the decision regarding the timing of surgery and supports early resection.

The ECCO consensus mentioned above states that ileocaecal CD should be treated by surgery only in patients with obstructive symptoms but no active inflammation. Based on our data, we suggest* early* resection in ileocaecal CD for demonstrable improvement in biochemical parameters, a notion which is supported in the literature [17]. More study is needed with a larger patient population, to study the effect on improvements to the nutritional status and also to comparatively assess the long-term outcomes of biologic therapy versus early surgical intervention. Furthermore, a long-term study examining the* subjective* improvement in terms of patients' quality of life would add to the study.

## Figures and Tables

**Table 1:** Summarised demographic data regarding patients in the study sample.

	Average	Maximum	Minimum
Age	28.2	47	16
Disease duration	19.8	38	2
Time between diagnosis and surgery	6.4	13	1
Follow-up	13.5	24	2

**Table 2:** Absolute values of biochemical parameters.

Absolute values	CRP	ESR	Hb	WBC
Average preop *(max/min)*	113.6 *(387/3.4)*	55.7 *(105/26)*	10.1 (*12.7/6.8)*	10245 *(16700/5610)*
Average postop *(max/min)*	21.8 *(114/2.2)*	42.7 *(112/10)*	11.1 *(15.6/7.46)*	7849 *(12800/4500)*
Average follow-up *(max/min)*	5.0 *(15/1.1)*	20.6 *(67/2)*	12.8 *(16.4/10.4)*	7144 *(10500/3740)*

**Table 3:** Percentage difference between preop to postop values and preop to follow-up values.

Average percentage improvement	CRP	ESR	Hb	WBC
Preop to postop (max/min)	71.5% (99.3/11.8)	36.9% (71.4/19.0)	12.2% (44.1/−3.9)	17.4% (40.3/−21.2)
Preop to follow-up (max/min)	85.5% (99.4/32.4)	64.3% (96.0/0.0)	29.3% (65.9/−3.6)	24.0% (51.0/−17.6)

**Table 4:** Summary table containing the means at the three data collection points, their standard deviations, and the relevant *F*-value and *p* value for each biochemical marker.

	CRP	ESR	Hb	WBC
Preop mean ± SD	113.5 ± 116.5	55 ± 33.9	10.0 ± 2.1	10245 ± 4459
Postop mean ± SD	21.8 ± 33.2	36.2 ± 27.0	11.1 ± 2.1	7849 ± 2496
Mean at follow-up ± SD	5.0 ± 4.7	18.5 ± 23.4	12.7 ± 2.2	7144 ± 2191
*F*-value	8.3	8.5	19.8	8.9
*p* value	0.013	0.019	<0.001	0.002

Patients with missing data are excluded from comparative analysis in a repeated measures ANOVA, thus explaining any disparities between means in Tables 4 and 2.
